# Low Prevalence of *Mycobacterium bovis* in Tuberculosis Patients, Ethiopia

**DOI:** 10.3201/eid2603.190473

**Published:** 2020-03

**Authors:** Muluwork Getahun, H.M. Blumberg, Waganeh Sinshaw, Getu Diriba, Hilina Mollalign, Ephrem Tesfaye, Bazezew Yenew, Mengistu Taddess, Aboma Zewdie, Kifle Dagne, Dereje Beyene, Russell R. Kempker, Gobena Ameni

**Affiliations:** Ethiopian Public Health Institute, Addis Ababa, Ethiopia (M. Getahun, W. Senshaw, G. Diriba, H. Mollalign, E. Tesfaye, B. Yenew, M. Taddess);; Addis Ababa University, Addis Ababa (M. Getahun, A. Zewdie, K. Dagne, D. Beyene, G. Ameni);; Emory University, Atlanta, Georgia, USA (H.M. Blumberg, R.R. Kempker)

**Keywords:** Bacteria, cattle, Ethiopia, livestock, *Mycobaterium bovis*, nontuberculous mycobacteria, PTB, pulmonary tuberculosis, tuberculosis and other mycobacteria, World Health Organization, zoonoses

## Abstract

An estimated 17% of all tuberculosis cases in Ethiopia are caused by *Mycobacterium bovis*. We used *M. tuberculosis* complex isolates to identify the prevalence of *M. bovis* as the cause of pulmonary tuberculosis. Our findings indicate that the proportion of pulmonary tuberculosis due to *M. bovis* is small (0.12%).

In 2016, the World Health Organization (WHO) estimated that there were 147,000 cases and 12,500 deaths worldwide from tuberculosis, which is predominantly caused by *Mycobacterium bovis.* However, because of the lack of comprehensive surveillance data, particularly from developing countries, actual illness and death could exceed this estimate (*1*,*2*). To enhance efforts at addressing zoonotic TB, multiple international organizations collaboratively developed and recently released the Roadmap for Zoonotic Tuberculosis (*1*). The roadmap states 3 objectives, the first of which is to collect more accurate scientific evidence on zoonotic TB through improved surveillance efforts. 

In Ethiopia, ≈80% of persons live in rural areas, where most of the population harvests crops or raises livestock (*3*). Because of the pastoral lifestyle, the burden of zoonotic TB is thought to be high in such rural communities because of a perceived higher risk of acquiring *M. bovis* infection (*2*). In 2013, Müller et al. estimated the proportion of all forms of TB cases caused by *M. bovis* in Ethiopia to be 17% (*4*). For this study, we evaluated the contribution of *M. bovis* toward causing pulmonary TB in Ethiopia. 

We obtained a total of 1,785 stored *M. tuberculosis* complex isolates collected from patients testing positive in smear tests. These tests were performed in 32 health facilities across Ethiopia during November 2011–June 2013 as part of a drug resistance survey. Among the 32 sites enrolled in the drug resistance survey, 30 sites had participated in an earlier survey in 2003–2005; two additional sites were selected from the Gambella and Benishangul Gumuz regions to ensure that >1 health facility from each region was included (Table). We included data from all patients with positive results for TB on consecutive sputum smear tests. 

**Table:** Number of cases by region and results of *Mycobacterium tuberculosis* testing for isolates in study of contribution of *M. bovis* to pulmonary tuberculosis, Ethiopia*

Region	Total no. cases	*M. tuberculosis* positive			*M. tuberculosis* negative	
		Culture positive	Culture negative/ contaminated		Culture positive	Culture negative/ contaminated
Addis Ababa	181	166	10		1	4
Afar	68	58	4		3	3
Amhara	138	121	6		5	6
Benishangul Gumuz	21	19	1		0	1
Dire Dawa	103	86	1		6	10
Gambella	121	105	4		7	5
Harar	52	50	2		0	0
Oromia	518	469	22		12	15
SNNPR	352	315	19		10	8
Somali	104	101	2		1	0
Tigray	77	62	10		2	3
Total	1,735	1,552	81		47	55

*Of the 1,735 isolates available for typing, 1,599 yielded positive results for *M. tuberculosis* complex; of those, 1,597 (99.87%) were *M. tuberculosis* positive by RD9 testing and 2 (0.13%) were *M. bovis* positive by RD4 testing. Of the 2 RD4 positive results, 1 was from culture-positive and the other from smear-positive (culture negative) sediment. RD, region of difference.

To identify species, region of difference (RD) 9- and RD4-based PCR procedures were performed using HVD primers and QIAGEN HotStarTaq Master Mix reagents (QIAGEN, https://www.qiagen.com), which were described in earlier studies (*5*–*8*). The Capilia TB-Neo test (Goffin Molecular Technologies, http://www.goffinmoleculartechnologies.com) was used to distinguish *M. tuberculosis*–complex species from other nontuberculous mycobacterial (NTM) species. The same standard operating procedures were used to interpret the results. 

Of the 1,785 isolates collected, 1,735 were available for typing. Among those typed, 1,599 (92%) yielded visible bands of *M. tuberculosis* complex. RD9 typing identified 1,597 (99.87%) of 1,599 isolates as *M. tuberculosis*, and RD4 typing identified only 2 (0.13%) of 1,599 of the isolates as *M. bovis*. These findings indicate that pulmonary TB due to *M. bovis* is rare in Ethiopia. 

This study has certain limitations. We used *M. tuberculosis* complex isolates collected from a sentinel drug resistance survey. Data from smears testing negative for pulmonary TB cases, which account for some proportion of PTB and extrapulmonary TB cases, were not included. 

One possible alternative explanation for finding few cases of *M. bovis* as a pathogen in pulmonary TB is that *M. bovis* may have been acquired through ingestion of food from livestock infected with extrapulmonary TB (*7*). In that case, sputum might not have been the ideal technique for isolating *M. bovis* samples. Previous studies in Ethiopia reported variable (0%–16%) prevalence of *M. bovis* in extrapulmonary TB patients (*8*,*9*). A second possible reason could be the low prevalence of bovine TB in zebu cattle, which comprise >95% of the cattle population of Ethiopia (*10*) and have been reported to have lower infection rates with *M. bovis* than other types of cattle. In addition, most cattle husbandry in Ethiopia is on extensively managed small farms in open fields, which poses a low risk for the spread of bovine TB (*7*). Thus, a low prevalence of bovine TB in the Ethiopia cattle population could result in a limited rate of transmission to humans. 

This study included samples from all regions of Ethiopia to identify the prevalence of bovine TB among patients with pulmonary TB. We found that *M. bovis* was an etiologic agent of human pulmonary TB in only a small fraction of cases, a lower proportion than previously estimated. This finding indicates that aerosol transmission of *M. bovis* from livestock to humans is rare. A useful focus for future efforts might be to implement or strengthen pasteurization programs in *M. bovis*–prevalent areas to limit possible transmission of bovine TB through the consumption of dairy products. 
